# Identification of a novel PD-L1 positive solid tumor transplantable in HLA-A*0201/DRB1*0101 transgenic mice

**DOI:** 10.18632/oncotarget.16900

**Published:** 2017-04-06

**Authors:** Laurie Rangan, Jeanne Galaine, Romain Boidot, Mohamad Hamieh, Magalie Dosset, Julie Francoual, Laurent Beziaud, Jean-René Pallandre, Elodie Lauret Marie Joseph, Afag Asgarova, Christophe Borg, Talal Al Saati, Yann Godet, Jean Baptiste Latouche, Séverine Valmary-Degano, Olivier Adotévi

**Affiliations:** ^1^ University Bourgogne Franche-Comté, INSERM, EFS BFC, UMR1098, Interactions Hôte-gGreffon-Tumeur, Ingénierie Cellulaire et Génique, F-25000 Besançon, France; ^2^ LabEx LipSTIC, F-25000 Besançon, France; ^3^ Platform for Transfer to Cancer Biology, Centre Georges-François Leclerc, 21000 Dijon, France; ^4^ University Hospital of Rouen, INSERM UMR1245, Institute for Research and Innovation in Biomedicine, 76183 Rouen, France; ^5^ Department of Medical Oncology, University Hospital of Besançon, 25000 Besançon, France; ^6^ INSERM/UPS, US006/CREFRE, Department of Histopathology, University Hospital of Purpan, 31000 Toulouse, France; ^7^ Department of Genetics, University Hospital of Rouen, Normandy Centre for Genomic and Personalized Medicine, 76183 Rouen, France; ^8^ Department of Pathology, University Hospital of Besançon, 25000 Besançon, France

**Keywords:** HLA transgenic mouse, PD-L1, sarcoma, T cells, cancer immunotherapy

## Abstract

HLA-A*0201/DRB1*0101 transgenic mice (A2/DR1 mice) have been developed to study the immunogenicity of tumor antigen-derived T cell epitopes. To extend the use and application of this mouse model in the field of antitumor immunotherapy, we described a tumor cell line generated from a naturally occurring tumor in A2/DR1 mouse named SARC-L1. Histological and genes signature analysis supported the sarcoma origin of this cell line. While SARC-L1 tumor cells lack HLA-DRB1*0101 expression, a very low expression of HLA-A*0201 molecules was found on these cells. Furthermore they also weakly but constitutively expressed the programmed death-ligand 1 (PD-L1). Interestingly both HLA-A*0201 and PD-L1 expressions can be increased on SARC-L1 after IFN-γ exposure *in vitro*. We also obtained two genetically modified cell lines highly expressing either HLA-A*0201 or both HLA-A*0201/ HLA-DRB1*0101 molecules referred as SARC-A2 and SARC-A2DR1 respectively. All the SARC-L1-derived cell lines induced aggressive subcutaneous tumors in A2DR1 mice *in vivo*. The analysis of SARC-L1 tumor microenvironment revealed a strong infiltration by T cells expressing inhibitory receptors such as PD-1 and TIM-3. Finally, we found that SARC-L1 is sensitive to several drugs commonly used to treat sarcoma and also susceptible to anti-PD-L1 monoclonal antibody therapy *in vivo*. Collectively, we described a novel syngeneic tumor model A2/DR1 mice that could be used as preclinical tool for the evaluation of antitumor immunotherapies.

## INTRODUCTION

The presence of a competent immune system, whereby tumor antigens are recognized as foreign and eliminated, is fundamental to the prevention of cancer development and progression. Molecular identification of tumor rejection antigen has helped define several classes of antigen. To evaluate the immunogenicity of tumor antigen-derived T cell epitopes *in vivo*, various HLA class I or HLA class II transgenic mouse models have been developed [1–5].

Among these mouse models, Lemonnier laboratory has created the new generation of humanized HLA-transgenic mice like the HLA-A*0201/DRB1*0101 (A2/DR1) mouse model. These mice are H-2 class I and IA class II knockout, and their CD8^+^ and CD4^+^ T cells are restricted by the sole HLA-A*0201 and HLA-DR1*0101 molecules, respectively [6]. According to the high frequency of these HLA alleles in the world population [7], this mouse model gained considerable interest in the field of tumor immunology. We and others previously used it for the identification and immunogenicity evaluation of T cell epitopes derived from many tumor antigens such as telomerase, Her-2/neu and NY-ESO-1 [8–12]. However, the use of these A2/DR1 mice is limited by the absence of suitable tumor models to evaluate the ability of tumor-derived epitopes to promote tumor rejection. Indeed, only non-syngeneic tumor cell lines engineered to express tumor antigens and HLA molecules were commonly used in these HLA transgenic mice [8, 13–15]. As these cell lines still express endogenous H2 class I and II molecules, the induction of non-specific T responses *in vivo* could not be excluded. This represents an important bias in the context of antitumor T cell response study. Considering these limitations, Schumacher et al. recently used a syngeneic tumor derived from a 3-methylcholantrene-induced sarcoma in A2/DR1 mouse model [11]. Here we described a non-chemical-induced tumor cell line derived from a spontaneously arising tumor in an A2/DR1 mouse named SARC-L1. This tumor cell line presents histological and genomic features consistent with a sarcoma and induces high aggressive tumors *in vivo*. In addition, SARC-L1 tumor cells express programmed death-ligand 1 (PD-L1) and the tumor microenvironment is highly infiltrated by T cells. Taken together, these results support the potential use of SARC-L1 tumor model for the evaluation of T cell based anticancer immunotherapies in A2/DR1transgenic mice.

## RESULTS

### Characterization of a novel syngeneic sarcoma tumor in HLA-A*0201/HLA-DR*0101 transgenic mice

**Figure 1:**
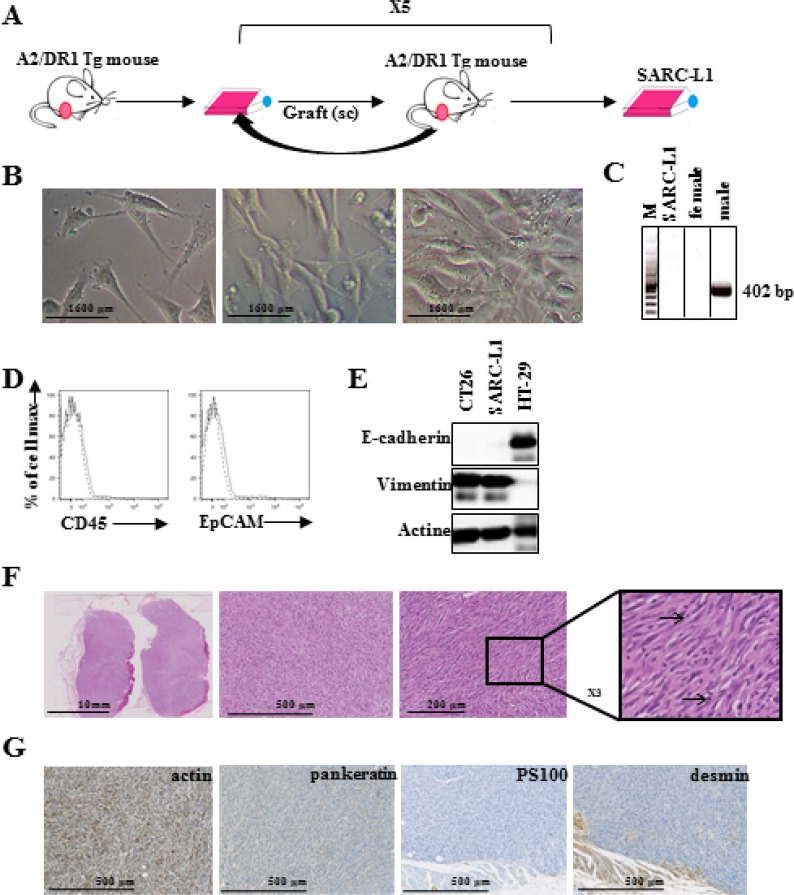
Novel transplantable sarcoma cell line in A2/DR1 mice (**A**) Generation of the mouse sarcoma cell line derived from a naturally occurring tumor in a 23- months-old female A2/DR1 mouse. (**B**) contrast microscopy aspects are shown. (**C**) Sry male-specific amplification from SARC-L1 cell line, and from female and male tissues by PCR. lane 1: 100 bp marker, lane 2: SARC-L1 DNA, lane 3: female DNA, lane 4: male DNA. (**D**) CD45 and EpCAM surface expression (solid line), and isotype (dotted line). (**E**) E-cadherin and vimentin expression by western blot analysis from SARC-L1, HT-29 (epithelial control) and CT26 (mesenchymal control). (**F**) Morphological analysis (hemalun and eosin staining) of SARC-L1 tumor. (**G**) Immunohistochemistry on SARC-L1 tumor with a positive staining for anti-alpha-smooth muscle actin antibody (left) and a negative staining for anti-pankeratin (middle left), anti-PS100 (middle right), anti-desmin (right).

This novel tumor cell line was generated from naturally spontaneous tumor appeared in a 23-months-old A2/DR1 mouse as described in material and method. Tumor was filtered and cell line was obtained after long term *in vitro* culture and serial transplantation (Figure 1A). In culture dish, SARC-L1 cell line appears fusiform shaped with long cytoplasmic extensions and is adherent cell line (Figure 1B). Its female origin was confirmed by the absence of amplification of *Sry* gene (Figure 1C). Phenotypical analysis showed that this cell line lacks the expression of leucocyte common antigen CD45 supporting its non-hematopoietic origin. SARC-L1 expressed neither epithelial cell marker E-cadherin nor the cell adhesion molecule EpCAM, but expressed the mesenchymal marker vimentin (Figure 1D and 1E).

The histological analysis showed that the nodular tumor is composed of spindle-shaped cells organized in intersecting long fascicles with a chevron-like pattern. Few foci of short fascicles with storiform pattern were found (Figure 1F). The cells have long nuclei with rounded ends and the stroma was poor with few collagen fibers. The presence of inflammatory cells such as lymphocytes and histiocytes was also found in the tumor microenvironment (TME) (Figure 1F right panel). We found intensive expression of the alpha-smooth muscle actin and absence of desmin, PS100 and cytokeratin (Figure 1G). Thus the morphological aspect of spindle cells and the expression of alpha-smooth muscle actin without epithelial or melanocytic markers support a sarcoma origin.

**Figure 2:**
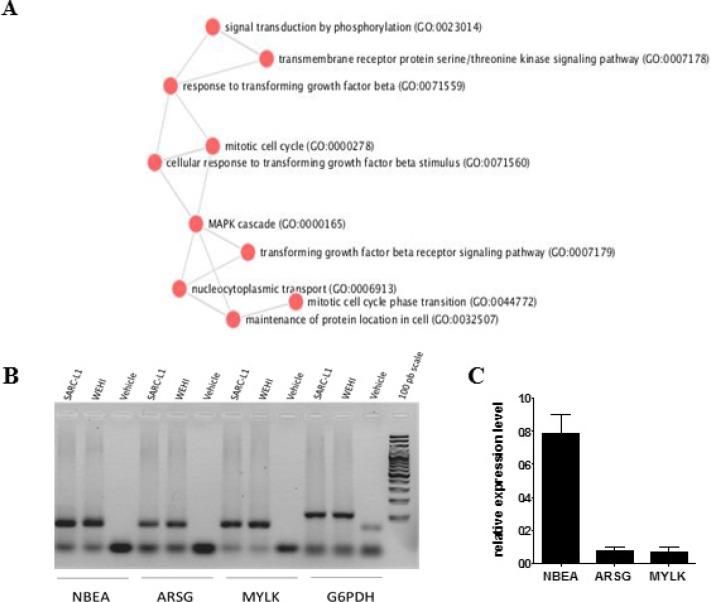
Genes expression profiling of SARC-L1 (**A**) Representative cluster of significant enrichment of biological processes obtained with Enrichr website. MAPK pathway, cell cycle processes and TGFβ response are highly activated in this cell line. B, C 3 related sarcoma genes (NBEA, ARSG, MYLK) were quantified by real-time quantitative PCR (**B**) Electrophoresis was performed with the products from the RTqPCR in a 2% agarose gel. (**C**) Relative expression of NBEA, ARSG and MYLK genes of SARC-L1 cell using WEHI-164 cells as gene of reference. The mRNA transcripts were calculated using the 2^ΔΔCt method. Amplification of the samples was performed in duplicate and the G6PDH mRNA transcript was used as housekeeping gene of reference (three independent experiments).

Genes expression profiling of SARC-L1 was performed using RNAsequencing. Nearly 3500 gene transcripts were detected. The analysis of molecular pathways involved using Enrichr website (http://amp.pharm.mssm.edu/Enrichr [16]) revealed statistically significant enrichment in different clusters of genes (Figure 2A). One of these clusters indicated that SARC-L1 cells harboured a strong activity in the RAS/MAPK pathway. Indeed, expressions of KRAS, BRAF, RAF1, MAP2K1, MAP2K2, MAPK1, MAPK7, MAPK12, CCM2, RAB1, RAB1b, RAB2a, RAB3gap2, RAB10, RAB18, and RAB26 were detected and confirmed the sarcoma origin [17, 18]. Other pathways corresponding to cancer proliferating cells were also enriched such as cell cycle, response to TGF-β and signal transduction. Moreover, the detection by RT-PCR of specific sarcoma genes like ARSG, MYLK, and NBEA confirmed our assumption, even though the level of expression of ARSG and MYLK were lower than that of mouse sarcoma WEHI-164 cell line used as positive control (Figure 2B and 2C). Thus, all these different molecular pathways identified SARC-L1 as a novel sarcoma-derived cell line.

### Effect of sarcoma-related cytotoxic drugs on SARC-L1

**Figure 3:**
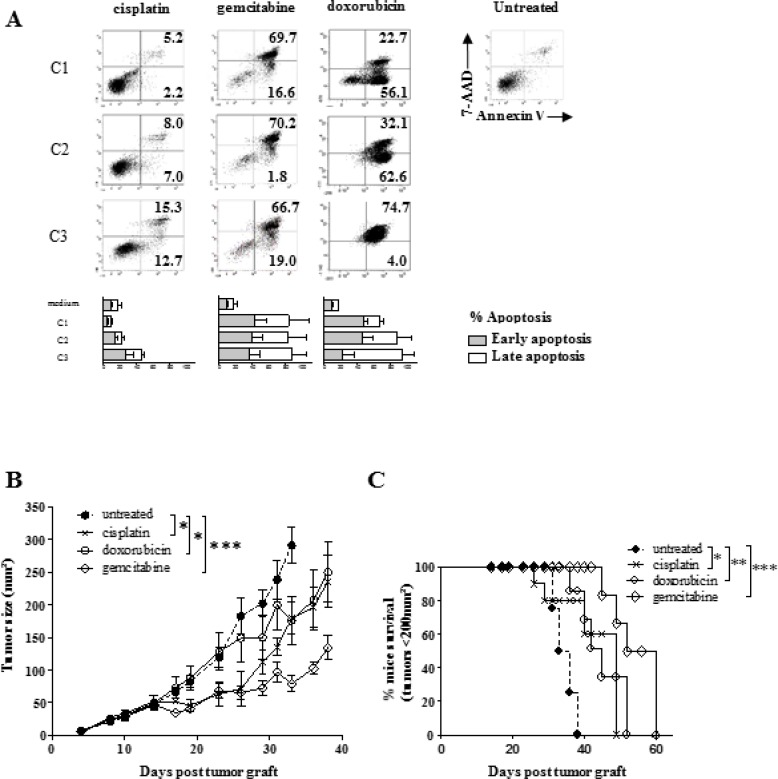
Effect of human sarcoma-related cytotoxic drugs on SARC-L1 cells (**A**) cells were cultured in presence of increasing doses of chemotherapies during 48 h and the percentage of apoptotic cells was measured using Annexin-V/7-AAD staining. Dot plots of SARC-L1 cells and percentage of early (Annexin-V+/7−AAD−) and late (Annexin-V+/7−AAD+) apoptotic cells. Dot plots are representative of three independent experiments. Bars represent means + SEM from the three independent experiments. (**B**) Average tumor size in the groups of A2/DR1-mice treated by i.p injection of gemcitabine 120 mg/kg, or cisplatin 7.5 mg/kg or doxorubicin 5 mg/kg, or saline (group control). Mean +/− SEM (*n* = 4–5 mice/group) *P < 0.05; ***P < 0.001 (Student test). (**C**) Kaplan–Meier curves. Log-rank (Mantel-Cox) tests are shown: *P < 0.05; **P < 0.01 ***P < 0.001.

The sarcoma origin prompted us to evaluate SARC-L1 sensitivity to various classes of cytotoxic drugs commonly used to treat human cancer such as platinum, antimetabolite, taxane, anthracyclin and alkylating agents. Cells were cultured in presence or not of increasing concentrations of each drug for 48 hours and cell apoptosis was measured by annexin-V/7-AAD staining as detailed in material and method. In contrast to platinum and taxane, antracyclin and antimetabolite agents induced a high rate of cell apoptosis (Figure 3A). The cell apoptosis induced by the antracyclin (doxorubicin or epirubicin) and the alkylating agent (dacarbazine) was dose-dependent. The antimetabolite (gemcitabine or methotrexate) induced significant cell death at low concentration *in vitro* (Figure 3A and Table 1). We next evaluated the cytotoxic drugs effects against SARC-L1 *in vivo*. To this end SARC-L1 bearing-A2/DR1 mice were treated with the indicated drugs. We observed that all these drugs induced a delay of SARC-L1 tumor growth but not complete tumor regression (Figure 3B). Similarly to the *in vitro* study, gemcitabine was found more effective against SARC-L1 *in vivo* as compared to cisplatin or doxorubicin (Figure 3B and 3C). Thus, SARC-L1 is susceptible to most sarcoma-related chemotherapies.

**Table 1:** SARC-L1 sensitivity to Cytotoxic drugs *in vitro*

	Drugs	% cell apoptosis (Annexin-V+)		
		C1	C2	C3
Antimetabolite	Gemcitabine	83.2 ± 10.3	82.6 ± 12.2	87.2 ± 9.2
	Methotrexate	49.9 ± 35.3	80.8 ± 2.8	86.6 ± 5.0
Platinum-based drugs	Cisplatin	8.6 ± 2.7	22.1 ± 7.6	45.8 ± 18.1
	Oxaliplatin	10.8 ± 8.9	26.6 ± 30.2	40.8 ± 30.1
Taxane	Docetaxel	37.0 ± 24.9	45.1 ± 24.6	47.0 ± 26.1
	Paclitaxel	15.5 ± 12.9	37.7 ± 22.2	46.9 ± 29.8
Anthracycline	Doxorubicin	66.5 ± 13.4	87.3 ± 13.1	90.6 ± 10.4
	Epirubicin	85.7 ± 12.1	86.8 ± 12.5	85.7 ± 6.8
Alkylating agents	Dacarbazine	39.2 ± 39.8	49.9 ± 32.5	82.3 ± 13.45

Drug concentrations were detailed in Material and Method section. Data represent mean ± SEM of percentage of cell apoptosis (Annexin-V+ cells) induced by different cytotoxic drugs at three increasing concentrations. Results represent at least three independent experiments.

### High T cell infiltration within SARC-L1 tumors

**Figure 4:**
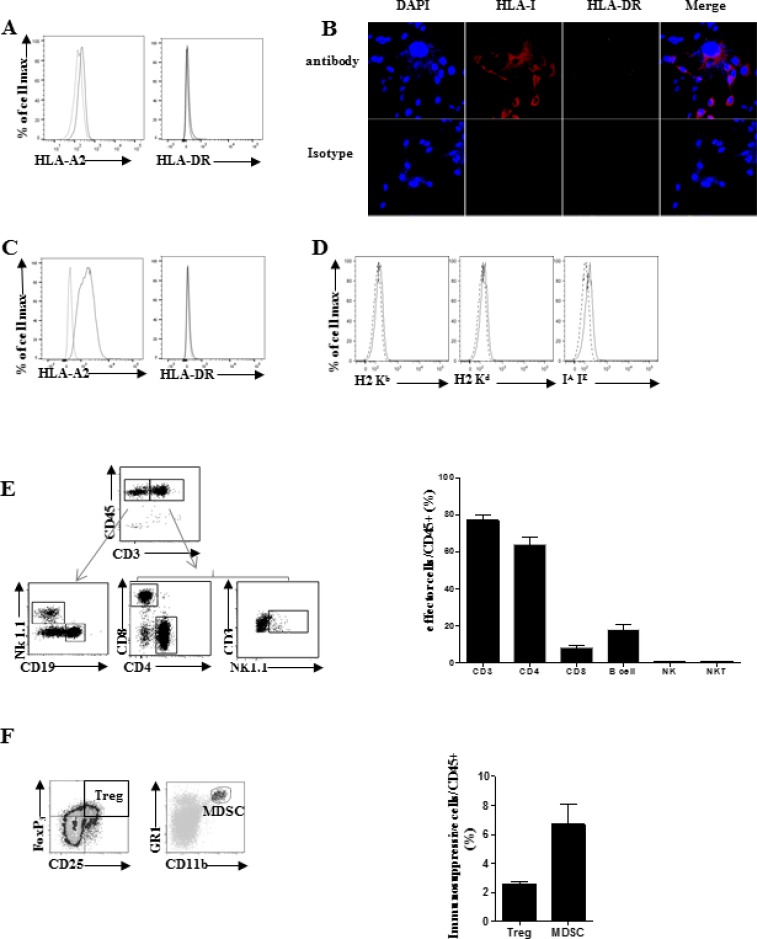
Analysis of MHC molecules expression by SARC-L1 cell line (**A**–**D**) Representative MHC molecules expression by SARC-L1. Human MHC (HLA-A2, and HLA-DR) are shown by flow cytometry (A) and confocal microscopy (B). Expression of HLA-A2 and HLA-DR on SARC-L1 cell line cultured *in vitro* in presence of murine recombinant IFN-γ (100 ng/ml) (D) Representative mouse murine MHC molecules expression by SARC-L1 (H2 Kb, H2 Kd, IA/IE). Specific markers (black line), isotype control (dotted line). (**E**, **F**) Tumor microenvironment of SARC-L1 in A2/DR1 mice were analyzed among CD45 positive tumor infiltrating cells. Gating strategy (left), histogram (right) represents mean of percentage +/− SEM (bar) ofimmune cells subpopulation (*n* = 10 mice). Effector cells (E) and immunosuppressive cells (F) Results are representative of three experiments analysed independently.

We first study the MHC molecules expression on SARC-L1 by using flow cytometry and confocal microscopy. While low expression of HLA-A2 molecule was observed on SARC-L1, this cell line lacks HLA-DR expression (Figure 4A and 4B). Similar low expression of HLA-A2 was found *ex vivo* on freshly isolated tumor cells from A2/DR1 mice (data not shown). Interestingly, HLA-A2 expression but not HLA-DR expression increased after IFN-γ exposure *in vitro* (Figure 4C). As expected, SARC-L1 did not express H-2K^b^, H-2K^d^ and IA/IE molecules supporting A2/DR1 mouse origin (Figure 4D).

To study the composition of immune cell infiltrate within SARC-L1 tumors, we performed flow cytometry analysis of tumors freshly isolated from A2DR1 mice. We showed that the microenvironment of SARC-L1 was highly infiltrated by CD3^+^ T cells which represents 76.8% of CD45^+^ TILs (Figure 4E). Among them the CD4 and CD8 T cell subsets represented 64.0% ± 3.5 % and 8.12 % ± 0.9 % of tumor infiltrating lymphocytes (TILs) respectively. The difference between the percentage of tumor-infiltrating CD4 and CD8 T cell is consistent with that commonly found in the spleen of tumor-free A2DR1 mice (data not shown). The B cells (CD45^+^CD3^−^CD19^+^) represented 18.1% ± 2.6% of TILs. In contrast NK cells (CD3^−^NK1.1^+^) and NKT cells (CD3+NK1.1+) were marginally represented (less than 1% of CD45^+^ cells) (Figure 4E). Next, we analyzed the immunosuppressive cells such as regulatory T cells (T_regs_) and myeloid derived-suppressive cells (MDSC) within the TME. As shown in Figure 4F, T_regs_ (CD45^+^CD4^+^CD25^+^FoxP_3_^+^ 2.5% ± 0.2%) and MDSCs (CD45^+^CD11b^+^Gr1^+^, 6.4% ± 1.3%) were detected within TME. Collectively SARC-L1 cells naturally express low level of HLA molecules and its tumor microenvironment is highly infiltrated by T cells.

### IFN-γ inducible PD-L1 expression on SARC-L1

**Figure 5:**
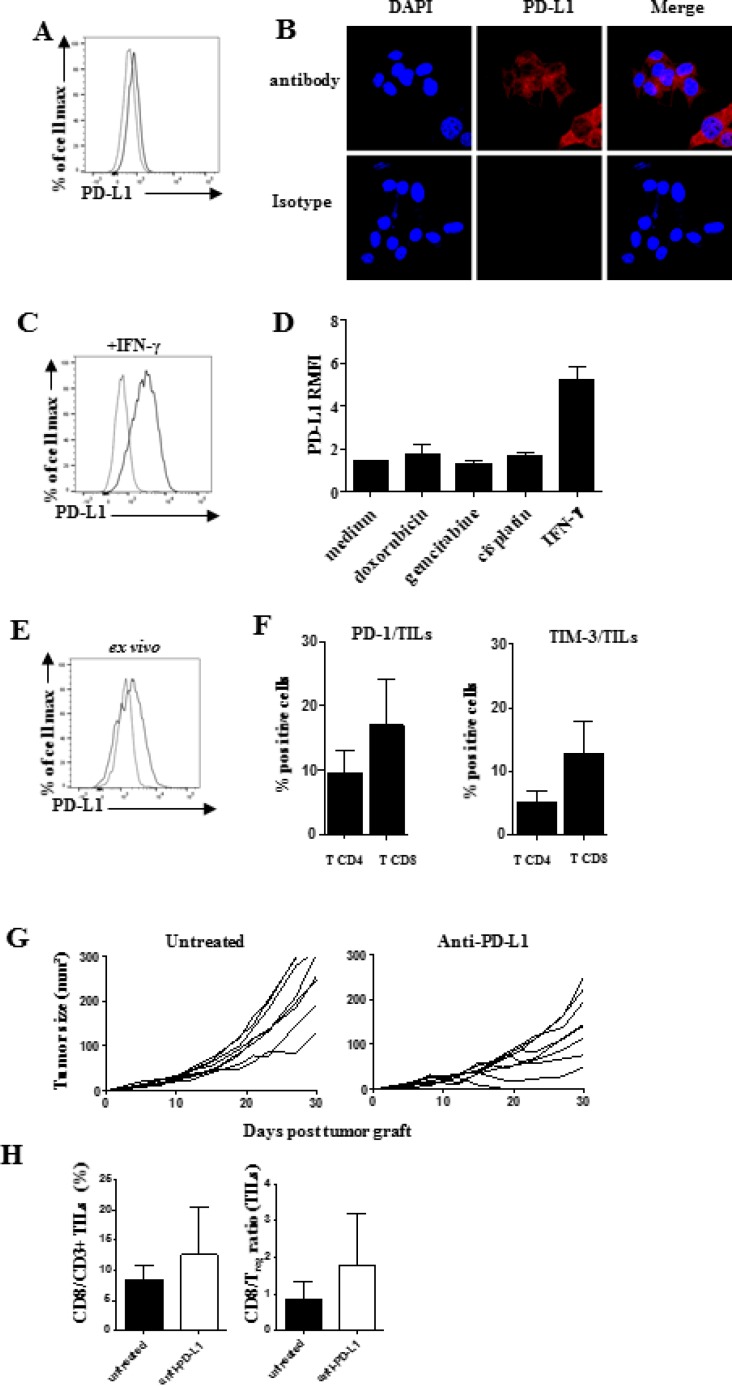
Presence of PD-1/PD-L1 axis in SARC-L1 tumor microenvironment A-B PD-L1 expression on SARC-L1 by flow cytometry (**A**) and confocal microscopy (**B**, **C**) PD-L1 expression on SARC-L1 by flow cytometry. SARC-L1 cells were cultured in presence of recombinant mIFN-γ or (**D**) indicated chemotherapies during 24 h (**E**) PD-L1 expression on *ex vivo* freshly isolated tumor cells from A2/DR1 mouse (**F**) PD-1 and TIM-3 expression. Representative histogram (right) in percentages +/− SEM (bar) of PD-1+ or TIM-3+ among CD4+ and CD8+ TILs cells (*n* = 10 mice) (**G**) Individual tumor size in SARC-L1 tumor bearing mice (*n* = 8 mice/group) treated with anti-PD-L1 or isotype control (untreated). Mice were treated or not by 4 × 200 μg anti-PD-L1 (*n* = 4–5 mice). (**H**) Percentage of CD8+ CD3+ TILs (left), CD8+ TIL/Treg ratio (right) in each group. Histogram represent mean + SEM.

The programmed death-ligand1 PD-L1 expression is a dominant mechanism used by tumor cells to escape from the T cells attack. Its expression is constitutively driven by aberrant oncogenic pathways or by a process named adaptive immune resistance that involves IFN-γ [19, 20]. Then, we investigated the PD-L1 expression by flow cytometry and found a constitutive but low expression of this immune checkpoint on SARC-L1 (Figure 5A). This result was also confirmed by confocal microscopy (Figure 5B). As shown in Figure 5C, PD-L1 expression on SARC-L1 is increased upon IFN-γ exposure *in vitro*, suggesting it may be induced by adaptive immunity *in vivo* [20]. It has been reported that PD-L1 could be induced on tumor cells upon treatment with chemotherapeutic agents that induce cell death signaling *in vitro* [21]. However cytotoxic drugs such as doxorubicin, gemcitabine and cisplatin did not influence PD-L1 expression on SARC-L1 (Figure 5D).

To assess whether PD-L1 could interact *in vivo* with the inhibitory receptor programmed-death (PD-1), we analyzed both PD-L1 and PD-1 expressions within the SARC-L1 TME. Similarly to *in vitro* experiments, we found a weak level of PD-L1 expression on tumor cells freshly isolated from A2/DR1 mice (Figure 5E). As shown in Figure 5F both CD4^+^ and CD8^+^ TILs expressed PD-1. Moreover the expression of TIM-3, another inhibitory receptor involved in T cell exhaustion, was detected on CD4^+^ and CD8^+^ TILs (Figure 5F). Given the presence of a PD-1/PD-L1 axis in SARC-L1 TME, we assessed the antitumor effect of therapy using an anti-PD-L1 blocking antibody. As depicted in Figure 5G, anti-PD-L1 treatment can inhibit tumor progression in SARC-L1 tumor-bearing mice. Moreover this therapy was associated with an increase of CD8^+^ TILs and CD8^+^TIL/Treg ratio (Figure 5H). Thus, the presence of PD-L1 expression on SARC-L1 makes this cell line targetable by PD1/PD-L1 pathway inhibitors in A2DR1 mice.

### Engineering SARC-L1 to overexpress HLA-A2 and HLA-DR molecules

**Figure 6:**
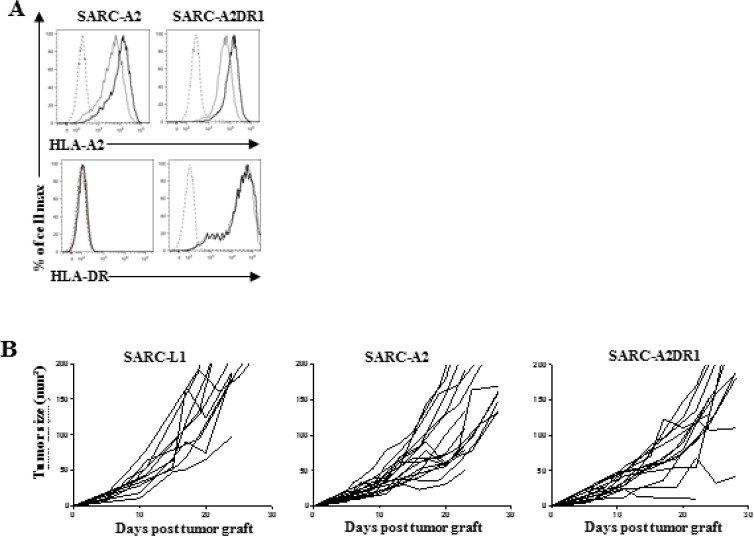
Transduction of SARC-L1 with syngeneic MHC-Class I and II molecules as potential tools to improve tumor-specific immunity model (**A**) MHC-Class I (HLA-A2.1) and MHC-Class II (HLA-DR.1) expression on SARC-A2 and SARC-A2DR1 tumor cells cultured overnight in the presence or not of recombinant mouse IFN-γ. (**B**) Tumor size measurement in A2/DR1 mice engrafted with 2.105 SARC-L1, SARC-A2 or SARC-A2DR1 cells (*n* = 10–17 mice).

To optimize the use of SARC-L1 in the context of T cell-based anticancer immunotherapies, we engineered SARC-L1 cells to co-express HLA-A2.1 and HLA-DR1 molecules. To this end we transduced them with gamma retroviral vectors encoding HLA-A2.1 and HLA-DR1. We obtained two genetically modified cell lines expressing HLA-A2.1 or both HLA-A2.1 and HLA-DR1 molecules, referred to as SARC-A2 and SARC-A2DR1 respectively. These cell lines expressed higher level of the two HLA molecules than parental SARC-L1 cell line (Figure 6A). Like for wild-type SARC-L1 cells, PD-L1 expression was also inducible by mIFN-γ on these cell lines (data not shown). Next, tumorigenicity of SARC-A2 and SARC-A2DR1 was investigated in A2/DR1 mice. As expected these cell lines were able to induce tumors after engraftment. However a delay of tumor growth was observed especially with SARC-A2DR1 as compared to SARC-L1 cell line (Figure 6B). Furthermore the composition of immune infiltrative cells was similar to the SARC-L1 tumor especially regarding the strong level of T cell infiltration.

Thus, SARC-A2 and SARC-A2DR1 cell lines could be a used as a preclinical tool to evaluate T cell-based immunotherapy in the context of HLA-A2 and HLA-DR1.

## DISCUSSION

In this study, we characterized a novel tumor cell line transplantable in A2/DR1 mouse named SARC-L1. This syngeneic tumor cell line was generated from a spontaneous tumor occurring in A2/DR1 transgenic mouse. Histological and genomic analysis revealed the sarcoma origin of SARC-L1. This cell line shows a high expression of vimentin, indicating its mesenchymal phenotype and supporting the aggressiveness of tumor growth in mice [22, 23]. Subcutaneous engraftment induced an aggressive tumor in A2/DR1 mice. However, the ability of SARC-L1 cell line to induce spontaneous metastasis was not clearly studied and needs future investigations.

As expected SARC-L1 did not express HLA DR molecules on cell surface [24, 25] but express low level of HLA-A2. Consequently, we speculate that the poor expression of HLA molecules on SARC-L1 cells allow them to escape from adaptive immunity. Interestingly HLA-A2 expression on SARC-L1 was increased after IFN-γ exposure, suggesting a quantitative abnormality of the HLA class I pathways as previously described in sarcoma [26, 27]. This overexpression of HLA-A2 could favor the recognition of SARC-L1 by CD8^+^ TILs cells within the tumor bed. In this line, engineering SARC-L1 to highly express HLA-A2 and HLA-DR1 molecules obviously increase its immunogenicity. This is exemplified by the delay of tumor growth observed in some A2/DR1 mice engraft mainly with SARC-L1 co-expressing both HLA-A2 and HLA-DR molecules. Like the parental SARC-L1, high T cell infiltration was found in SARC-A2 and SARC-A2DR1 tumors. Thus, these cell lines represents a suitable tumor model to evaluate the efficiency of antitumor immunotherapy in the context of HLA-A2 and HLA-DR1 restriction.

This last decade, the role of PD-1/PD-L1 axis has been extensively investigated in several cancers. This pathway represents a major immune escape mechanism developed by many tumors [28, 29]. We observed on SARC-L1 a low level of PD-L1 expression which is highly increased after IFN-γ exposure. This suggests that both constitutive oncogenic and adaptive immune resistance mechanisms can drive PD-L1 expression on this cell line [28]. Although a strong activity in the RAS/MAPK pathway was found in SARC-L1 cell line, the association between oncogenic driver mutations and PD-L1 expression has not been explored in this study. Then we believe that the PD-L1 induction by IFN-γ might create a barrier against effector CD8 TILs attack [19, 28, 30]. The importance of PD-1/PD-L1 interaction in this tumor model is further supported by the ability of anti-PD-L1 therapy to delay SARC-L1 tumor growth and to increase CD8^+^ TILs infiltration.

In conclusion, we described a novel non chemical-induced sarcoma tumor model (SARC-L1) from A2/DR1 mice. This syngeneic tumor model is suitable to investigate tumor antigens immunogenicity in the context of HLA-A2 and HLA-DR1 restriction. The presence of PD1/PD-L1 axis in SARC-L1 also offers an attractive tool to evaluate immune checkpoint inhibitors and combinations approaches.

## MATERIALS AND METHODS

### Mice

HLA-DRB1*0101/HLA-A*0201 transgenic mice (A2/DR1 mice) have been previously described [6]. Mice were purchased at the production from the center of “Cryopreservation, Distribution, Typage et Archivage Animal”. Male or female mice aged of 6 to 10 weeks were used in the experiments. All experiments were carried out according to the good laboratory practices defined by the animal experimentation Rules in France.

### Tumor cell line generation

A spontaneous subcutaneous tumor occurring in a 23-months-old female A2/DR1 transgenic mouse has been excised. Then tumor cells have been separated by filtration on 700 nm filter (Miltenyi, France) and a cell line was obtained after long term of *in vitro* culture. To generate a syngeneic cell line transplantable in A2/DR1 mouse, this cell line was subcutaneously injected into the flanks of female A2/DR1 mouse and the cell line was derived from successful tumor graft. This protocol has been repeated five times to have a reproductive tumor growth at 2.10^5^ cells both in female and male mice (Figure 1A). The tumor cell line generated was cultured in RPMI 1640 (Gibco, France) supplemented with 10 % fetal calf serum (Gibco, France) and 1% penicillin/streptomycin (Gibco, France)

### Morphological and immunohistochemical analysis

Tissue samples were fixed in 4% formalin and were paraffin-embedded. Four micrometer routinely processed paraffin sections were performed for standard coloration by hemalun and eosin and for immunohistochemistry using a VentanaBenchMark XT immunostainer (Ventana medical system, Tucson, Arizona). Immunostaining was performed with the following primary antibodies: anti-cytokeratin (Clone AE1/AE3, Ménarini, 1/400), anti-actin (Clone 1A4, Dako, 1/300), anti-desmin (Clone D33, Dako, 1/100) and anti-PS100 (polyclonal rabbit antibody, Leica, 1/400).

### Western blot

Cells were harvested and lysed as previously described [12]. The blots were incubated with specific antibodies as follows: E-cadherin, Vimentin, (Cell Signaling, Montigny-le-Bretonneux, France), β-actine (Sigma Aldrich, Saint Quentin, France). Blotted proteins were detected and quantified on a bioluminescence imager and BIO-1D advanced software (Wilber-Lourmat, France), after incubating blots with a horseradish peroxidase conjugated appropriate secondary antibody (Beckman Coulter, Villepinte, France).

### Genotyping and transcriptomic analysis

DNA from SARC-L1cell line was extracted by DNeasy Blood and Tissue Kit (Qiagen). With genomic DNA from tail biopsy, mice were genotyped by PCR for the sex determining Y region (*Sry*). PCR using a set of primers specific for the Sry male-specific primers pair generated a 402 bp length band in male derived DNA that was absent in female-derived DNA. The PCR primers used were 5′- ATGGAGGGCCATGTCAAG-3′ and 5′-AACAGGCTGCCAATAAAAGC-3′. For preparation of RNA-sequencing libraries, total RNA from cells was extracted with Trizol reagent. mRNA were purified with NEBNext Poly(A) mRNA magnetic module and used for the library preparation with a NEBNext Ultra RNA library kit for Illumina according to the manufacturer's instructions. RNA sequencing was performed on a MiSeq device. The libraries were sequenced with paired-end 75-base paired reads. More than 5 million reads were produced for each library. For gene expression profile of specific sarcoma genes (*ARSG, MYLK, NBEA*), real time quantitative polymerase chain reaction (qRT-PCR) (Thermofisher, France) was performed using primer sets listed as follows : *NBEA* (Mm01281997_g1), *ARSG* (Mm00546931_m1), *MYLK* (Mm00546931_m1) (Assay On Demand, Applied Biosystems). RT-qPCR was performed on the iCycler CFX96 realtime PCR system (Bio-Rad). Relative expression for the mRNA transcripts was calculated using the 2^−ΔΔCt^ method and *GAPDH* mRNA transcript as housekeeping gene of reference. The mouse sarcoma cell line WEHI-164 cell line was used as positive control.

### Flow cytometry

For the analysis of MHC molecules expression by SARC-L1, cells were stained with the following fluorescent conjugated monoclonal antibodies: Anti-2K^b^, (clone AF6-88.5.5.3,); anti-H-2K^d^and anti-I^A^/I^E^ (Ebiosciences, France); anti-HLA-A2 (BD Biosciences, France); anti-HLA-DR (Diaclone, France) or with their respectively isotype control. For tumor infiltrating immune cells analysis, tumor cells were mechanically dissociated (GentleMax, Miltenyi, France) tumor infiltrating lymphocytes were separated on a Percoll (Sigma-Aldrich, France) density gradient. Lymphocytes layer was collected and stained with the following fluorescent conjugated monoclonal antibodies: anti-CD3, anti-CD4, anti- CD8, anti-Gr1, anti-PD-L1 (Biolegend, France), anti-CD11b, anti- FoxP_3_, anti-CD25 (eBiosciences, France), anti-PD-1, and anti-TIM-3 (Miltenyi, France), fixable viability stain (BD, France). The tumor cells fraction was stained withanti-CD45, anti-PD-L1, anti-HLA-A2 and anti-HLA-DR (BD Biosciences, France) and fixable viability stain (Biolegend, France). Samples were acquired on a FACS Canto II (BD Biosciences, France) and analyzed with the BDFacsDIVA or FlowJo software.

### Confocal microscopy

HLA A2, HLA-DR and PD-L1 expression were evaluated by immunofluorescent staining. SARC-L1 were cultured overnight. Then were washed with PBS, fixed with 3.7% formaldehyde and washed with PBS 1% FBS. Cells were incubated 2 hours with 1/200 diluted primary antibodies: anti-HLA-class I (clone W6/32, Santacruz) and anti HLA-DR (clone YE2/36 HKL, Thermofisher) and stained with the secondary antibody during 2 hours (anti-mouse IgG2a AF594, Jackson; anti-rat IgG2a Dylight488, Thermofischer). For PD-L1 confocal staining, SARC-L1 were directly incubated with coupled anti-PDL1 antibody (clone 10F.9G2). Finally, cells were washed with PBS and stained with DAPI (Invitrogen) and mounted in Dako mounting medium. Samples were imaged using Olympus IX81 scanning confocal microscope.

### *In vitro* cell apoptosis induction with chemotherapies

SARC-L1 cells were cultured *in vitro* in presence of different class of cytotoxic drugs used against human cancer (platinum, antimetabolites, taxanes, anthracyclines and alkylating agents). For cell death analysis, SARC-L1 cells were cultured in 24-well plates (1 × 10^5^ cells per well)in presence or not of each drug at 3increasing concentrations according to previous studies. The following drugs (C1; C2; C3) were used: gemcitabine (4; 40; 200 nM), methotrexate (0.017; 0.17; 0.85 μM), cisplatine (0.2; 2; 10 μM), oxaliplatin (0.8; 8; 40 μM), docetaxel (10; 100; 500 μM), paclitaxel (2; 20; 100 μM), doxorubicine (1; 10; 50 μM), epirubicin (9; 90; 450 μM), and dacarbazine (0.8; 8; 40 mM). After 48 hours of culture, cell death was assessed by flow cytometry using Annexin-V and 7-AAD (BD, Biosciences France) according to the manufacturer's instructions. Samples were acquired on a FACS Canto II (BD Biosciences) and analyzed with the DIVA software. Results showed the percentage of early and late apoptotic cells (Ann^+^/7-AAD^−^ and Ann^+^/7-AAD^+^ respectively).

### Generation of HLA-A2.1/DR1-expressing SARC-L1 cell lines

SARC-L1 cells were transduced with gammaretrovial vectors encoding human b2-microglobulin and A*02.01 heavy chain to express HLA-A*02.01 (A2.1) complete molecule (SARC-A2), and with gammaretrovial vectors encoding DRα chain and DRβ1*01:01 chain to express HLA-DRB1*01:01 (DR1) complete molecule (SARC-A2DR1). Precise transduction procedures were already described [31, 32]. Briefly, each vector was transfected into H29/293 GPG packaging cells by Calcium Chloride precipitation method. Sarc-L1 cells were then transduced with the different cell-free gammaretroviral supernatants in the presence of Polybrene (Sigma-Aldrich, St. Louis, MO, USA), 8 μg/mL, for 16 hours. Finally, cells expressing A2.1 and DR1 were purified using anti-A2 antibody (BD Pharmingen, San Diego, CA, USA), anti-DR antibody (Caltag Laboratories, Burlingame, CA) and anti-mouse IgG-coated magnetic beads (Dynabeads, Dynal, Oslo, Norway), following the manufacturers' instructions.

### Tumor challenge and treatments

A2/DR1 mice were subcutaneously (s.c) injected with either 2.10^5^ SARC-L1, SARC-A2 or Sarc-A2DR1 cells in 100 μL of a saline solution in the abdominal flank. In all experiments, treatment started when tumor measured an average of 30–50 mm^2^. Tumor growth was monitored every 2 or 3 days using a caliper and mice were euthanized when their tumor size exceeded 300 mm^2^. All experiments were carried out according to the good laboratory practices defined by the animal experimentation rules in France. For chemotherapy, tumor-bearing mice were injected intraperitoneally (i.p) either with doxorubicin 5 mg/kg per week for 3 weeks, gemcitabine 120 mg/kg/week for 3 weeks or cisplatin 7.5 mg/kg/week for 2 weeks [33, 34]. Control mice received a saline solution i.p. Anti-mPD-L1Mab (clone10F.9G2, BioXcell) was injected i.p at 200 μg every 3–4 days four times.

### Statistical analysis

Data are presented as mean ± standard error SEM. Statistical comparison between groups was based on Student *t* test using Prism 6 GraphPad Software (San Diego, CA, USA). Mouse survival was estimated from the tumor size of 300mm^2^ by Kaplan–Meier method and the log-rank test. *P* values less than 0.05 were considered as statistically significant (*P < 0.05, **P < 0.01, ***P < 0.001).

## References

[1] Adotévi O, Mollier K, Neuveut C, Cardinaud S, Boulanger E, Mignen B, Fridman W-H, Zanetti M, Charneau P, Tartour E, Lemonnier F, Langlade-Demoyen P (2006). Immunogenic HLA-B*0702-Restricted Epitopes Derived from Human
Telomerase Reverse Transcriptase That Elicit Antitumor Cytotoxic T-Cell
Responses. Clin Cancer Res.

[2] Boucherma R, Kridane-Miledi H, Bouziat R, Rasmussen M, Gatard T, Langa-Vives F, Lemercier B, Lim A, Bérard M, Benmohamed L, Buus S, Rooke R, Lemonnier FA (1950). HLA-A*01: 03, HLA-A*24: 02, HLA-B*08: 01, HLA-B*27: 05, HLA-B*35: 01,
HLA-B*44: 02, and HLA-C*07: 01 monochain transgenic/H-2 class I null mice: novel
versatile preclinical models of human T cell responses. J Immunol.

[3] Pascolo S (2005). HLA class I transgenic mice: development, utilisation and
improvement. Expert Opin Biol Ther.

[4] Osen W, Soltek S, Song M, Leuchs B, Steitz J, Tüting T, Eichmüller SB, Nguyen X-D, Schadendorf D, Paschen A (2010). Screening of human tumor antigens for CD4 T cell epitopes by
combination of HLA-transgenic mice, recombinant adenovirus and antigen peptide
libraries. PloS One.

[5] Ru Z, Xiao W, Pajot A, Kou Z, Sun S, Maillere B, Zhao G, Ojcius DM, Lone Y, Zhou Y (2012). Development of a Humanized HLA-A2.1/DP4 Transgenic Mouse Model and the
Use of This Model to Map HLA-DP4-Restricted Epitopes of HBV Envelope
Protein. PLoS One.

[6] Pajot A, Michel M-L, Fazilleau N, Pancré V, Auriault C, Ojcius DM, Lemonnier FA, Lone Y-C (2004). A mouse model of human adaptive immune functions:
HLA-A2.1-/HLA-DR1-transgenic H-2 class I-/class II-knockout mice. Eur J Immunol.

[7] Gonzalez-Galarza FF, Christmas S, Middleton D, Jones AR (2011). Allele frequency net: a database and online repository for immune gene
frequencies in worldwide populations. Nucleic Acids Res.

[8] Dosset M, Godet Y, Vauchy C, Beziaud L, Lone YC, Sedlik C, Liard C, Levionnois E, Clerc B, Sandoval F, Daguindau E, Wain-Hobson S, Tartour E (2012). Universal cancer peptide-based therapeutic vaccine breaks tolerance
against telomerase and eradicates established tumor. Clin Cancer Res Off J Am Assoc Cancer Res.

[9] Gritzapis AD, Voutsas IF, Baxevanis CN (2012). Ontak reduces the immunosuppressive tumor environment and enhances
successful therapeutic vaccination in HER-2/neu-tolerant mice. Cancer Immunol Immunother CII.

[10] Johannsen A, Genolet R, Legler DF, Luther SA, Luescher IF (2010). Definition of key variables for the induction of optimal
NY-ESO-1-specific T cells in HLA transgene mice. J Immunol.

[11] Schumacher T, Bunse L, Pusch S, Sahm F, Wiestler B, Quandt J, Menn O, Osswald M, Oezen I, Ott M, Keil M, BalΔ J, Rauschenbach K (2014). A vaccine targeting mutant IDH1 induces antitumour
immunity. Nature.

[12] Vauchy C, Gamonet C, Ferrand C, Daguindau E, Galaine J, Beziaud L, Chauchet A, Henry Dunand CJ, Deschamps M, Rohrlich PS, Borg C, Adotevi O, Godet Y (2015). CD20 alternative splicing isoform generates immunogenic CD4 helper T
epitopes. Int J Cancer.

[13] Adotévi O, Mollier K, Neuveut C, Dosset M, Ravel P, Fridman W-H, Tartour E, Charneau P, Wain-Hobson S, Langlade-Demoyen P (2010). Targeting human telomerase reverse transcriptase with recombinant
lentivector is highly effective to stimulate antitumor CD8 T-cell immunity in
vivo. Blood.

[14] Gross D-A, Graff-Dubois S, Opolon P, Cornet S, Alves P, Bennaceur-Griscelli A, Faure O, Guillaume P, Firat H, Chouaib S, Lemonnier FA, Davoust J, Miconnet I (2004). High vaccination efficiency of low-affinity epitopes in antitumor
immunotherapy. J Clin Invest.

[15] Taieb J, Chaput N, Schartz N, Roux S, Novault S, Ménard C, Ghiringhelli F, Terme M, Carpentier AF, Darrasse-Jèze G, Darrasse-Jèse G, Lemonnier F, Zitvogel L (2006). Chemoimmunotherapy of tumors: cyclophosphamide synergizes with exosome
based vaccines. J Immunol.

[16] Kuleshov MV, Jones MR, Rouillard AD, Fernandez NF, Duan Q, Wang Z, Koplev S, Jenkins SL, Jagodnik KM, Lachmann A, McDermott MG, Monteiro CD, Gundersen GW (2016). Enrichr: a comprehensive gene set enrichment analysis web server 2016
update. Nucleic Acids Res.

[17] Baird K, Davis S, Antonescu CR, Harper UL, Walker RL, Chen Y, Glatfelter AA, Duray PH, Meltzer PS (2005). Gene expression profiling of human sarcomas: insights into sarcoma
biology. Cancer Res.

[18] Brohl AS, Solomon DA, Chang W, Wang J, Song Y, Sindiri S, Patidar R, Hurd L, Chen L, Shern JF, Liao H, Wen X, Gerard J (2014). The genomic landscape of the Ewing Sarcoma family of tumors reveals
recurrent STAG2 mutation. PLoS Genet.

[19] Topalian SL, Taube JM, Anders RA, Pardoll DM (2016). Mechanism-driven biomarkers to guide immune checkpoint blockade in
cancer therapy. Nat Rev Cancer.

[20] Pardoll DM (2012). The blockade of immune checkpoints in cancer
immunotherapy. Nat Rev Cancer.

[21] Galluzzi L, Buqué A, Kepp O, Zitvogel L, Kroemer G (2015). Immunological Effects of Conventional Chemotherapy and Targeted
Anticancer Agents. Cancer Cell.

[22] Tian W, Wang G, Yang J, Pan Y, Ma Y (2013). Prognostic role of E-cadherin and Vimentin expression in various
subtypes of soft tissue leiomyosarcomas. Med Oncol Northwood Lond Engl.

[23] Techasen A, Loilome W, Namwat N, Khuntikeo N, Puapairoj A, Jearanaikoon P, Saya H, Yongvanit P (2014). Loss of E-cadherin promotes migration and invasion of
cholangiocarcinoma cells and serves as a potential marker of
metastasis. Tumour Biol J Int Soc Oncodevelopmental Biol Med.

[24] Ostrand-Rosenberg S, Roby C, Clements VK, Cole GA (1991). Tumor-specific immunity can be enhanced by transfection of tumor cells
with syngeneic MHC-class-II genes or allogeneic MHC-class-I genes. Int J Cancer Suppl.

[25] Leach DR, Callahan GN (1995). Fibrosarcoma cells expressing allogeneic MHC Class II antigens induce
protective antitumor immunity. J Immunol.

[26] Maschek U, Pülm W, Segal S, Hämmerling GJ (1989). Major histocompatibility complex class I genes in murine fibrosarcoma
IC9 are down regulated at the level of the chromatin structure. Mol Cell Biol.

[27] Engel AM, Svane IM, Madsen MW, Pedersen M, Werdelin O (1997). Molecular aberrations in the MHC class I-restricted pathway for
antigen presentation in methylcholanthrene sarcomas from nude mice: discrepancies
between MHC mRNA and surface protein. Clin Exp Immunol.

[28] Pardoll DM (2012). The blockade of immune checkpoints in cancer
immunotherapy. Nat Rev Cancer.

[29] Vesely MD, Kershaw MH, Schreiber RD (2011). Natural Innate and Adaptive Immunity to Cancer. Annu Rev Immunol.

[30] Skoulidis F, Byers LA, Diao L, Papadimitrakopoulou VA, Tong P, Izzo J, Behrens C, Kadara H, Parra ER, Canales JR, Zhang J, Giri U, Gudikote J (2015). Co-occurring genomic alterations define major subsets of KRAS-mutant
lung adenocarcinoma with distinct biology, immune profiles, and therapeutic
vulnerabilities. Cancer Discov.

[31] Fauquembergue E, Toutirais O, Tougeron D, Drouet A, Le Gallo M, Desille M, Cabillic F, de La Pintière CT, Iero M, Rivoltini L, Baert-Desurmont S, Leprince J, Vaudry H (1997). HLA-A*0201-restricted CEA-derived peptide CAP1 is not a suitable
target for T-cell-based immunotherapy. J Immunother Hagerstown Md.

[32] Garnier A, Hamieh M, Drouet A, Leprince J, Vivien D, Frébourg T, Le Mauff B, Latouche J-B, Toutirais O (2016). Artificial antigen-presenting cells expressing HLA class II molecules
as an effective tool for amplifying human specific memory CD4(+) T
cells. Immunol Cell Biol.

[33] Ghiringhelli F, Apetoh L (2014). The interplay between the immune system and chemotherapy: emerging
methods for optimizing therapy. Expert Rev Clin Immunol.

[34] Hemmerle T, Probst P, Giovannoni L, Green AJ, Meyer T, Neri D (2013). The antibody-based targeted delivery of TNF in combination with
doxorubicin eradicates sarcomas in mice and confers protective
immunity. Br J Cancer.

